# Study of microcomb threshold power with coupling scaling

**DOI:** 10.1038/s41598-021-89411-0

**Published:** 2021-05-11

**Authors:** Pei-Hsun Wang, Kuan-Lin Chiang, Zong-Ren Yang

**Affiliations:** Department of Optics and Photonics, National Central University, Taoyuan City, 32001 Taiwan

**Keywords:** Nanoscience and technology, Optics and photonics

## Abstract

We model the generation threshold and conversion efficiency of microcombs by scaling the cavity coupling. With the Lugiato–Lefever equation (LLE), quantitative analysis of threshold is established in the parameter space of pump power and coupling. Considering the large detuning and Kerr-induced phase shift, the threshold power is numerically solved with the minimum at over-coupling, in agreement with that from the traveling wave theory. Furthermore, the coupling dependence on microcomb generation is discussed, providing the accessibility of high-efficient, stable combs (≥ 40%) around the threshold. This work offers universal guidelines for the design of microcombs with low-power and high-efficient operation.

## Introduction

Optical frequency comb generation in high quality factor (Q) microresonators has been widely studied within the past decades^1–5^. By pumping with continuous-wave (CW) in high-Q resonators through tapered fibers or a bus waveguide, cascaded four-wave-mixing (FWM) process is initiated when the nonlinear gain balances the cavity loss. The small, compact, and CMOS-compatible platform provides the potential for on-chip comb applications ranging from spectroscopy^6^ to telecommunications^7^. Microcomb generation has been extensively reported both theoretically and experimentally. Theoretically, through the Kerr nonlinearity, parametric gain induces modulation instability (MI) in the microresonators with the anomalous dispersion. The parametric gain is dependent on the cavity power in a parametric oscillator, showing a strong correlation with the cavity loss and coupling; low threshold power at sub-mW levels is observed in an ultrahigh-Q microcavity^8^. In numerical modeling, the optical system in microresonators can be described by a driven, detuned, and damped nonlinear Schrödinger equation^9^, which is also well-known as the Lugiato-Lefever equation (LLE)^9–12^. Steady-state solutions are explored in the region of cnoidal waves (Turing rolls) or dissipative solitons, relating to the pump detuning, amplitude of cavity fields, and waveguide dimensions^13,14^. However, although these reports based on the LLE provide significant insight into the generation dynamics for Kerr microcombs, the discussion on comb threshold with regard to the pump detuning, cavity loss, and coupling is still limited. Meanwhile, as for the conversion efficiency, the optimized comb state has been demonstrated through simulation trials over a parameter space consisting of group velocity dispersion (GVD), coupling, and pump detuning^15,16^. It was shown that only a few percent efficiency is modelled for a bright pulse in the anomalous dispersion while high efficiency is possibly achieved for a dark pulse in the normal dispersion. Experimentally, since the cavity pump saturates in the presence of frequency combs^17^, high conversion efficiency up to 30% can be observed with an initially over-coupled microcavity by employing dark pulses^18^; more recently, low-noise combs with conversion efficiency up to 41% is obtained using a coupled-ring geometry in the normal dispersion regim^19^. However, the conversion efficiency is mainly studied in the soliton regime, typically requiring high-power and delicate operation. There is still no clear guideline to design the coupling and initiate high efficient combs around the threshold with low-power operation. It now becomes crucial to address these issues and provide a pathway for the design of microresonator-based comb platform as a portable, battery-powered system^20^.

In this article, we theoretically investigate the relation between the coupling, pump power, parametric oscillation threshold, and conversion efficiency of combs by solving the generalized mean-field LLE. By slowly tuning the pump power in the cold cavity, the threshold and the comb dynamics are investigated under different couplings. Here, we consider the comb process in the anomalous dispersion region, in which MI in a continuously pumped resonator is the main generation mechanism of Kerr microcombs. The threshold we investigate in this article is the minimal input power for the parametric gain overcoming the cavity loss to induce cavity MI. This work results in several new findings. First, the correlation between the cavity coupling and comb threshold is built by the LLE. Considering the pump detuning and the nonlinear phase accumulated by the intracavity field, non-zero phase shifts result in an increase of the MI threshold power. The optimized coupling for minimal threshold moves from the under-coupling to over-coupling similar to that observed from the parametric threshold equation^8^. Through the LLE, this work qualitatively verifies the required MI power for cnoidal wave generation with a small detuning and the up-switching power for soliton generation with a large detuning. Second, the evolution of Kerr-comb formation near the threshold is discussed at different coupling regimes. Stable, high-periodic cnoidal waves boundaries are observed similar to that previously identified with different pump detunings^13^. In the meantime, this approach allows us to build the relationship between the coupling and the required switching power to initiate cavity solitons. Third, by utilizing the traveling wave theory, we comprehensively explore the threshold under the dimensionalities of detuning, coupling, and cavity loss. These analytical solutions yield the complemental results with that from the LLE. Last, the conversion efficiency of comb generation is discussed near the threshold. Stable cnoidal waves are identified in the regime of strongly over-coupling with high conversion efficiency, even up to 40% in the anomalous dispersion. Comparing with the complicated process for cavity soliton generation, cnoidal waves provide efficient, simple, and robust comb operation for potential applications such as in optical communications^21^.

## Method

We start our approach by numerically solving the generalized mean-field LLE, written as^12^:

1 $$ T_{R} \frac{\partial E}{{\partial t}} = [ - \frac{{\alpha_{i} + \theta }}{2} - i\delta_{0} + iL\sum\limits_{k \ge 2} {\frac{{\beta_{k} }}{k!}(i\frac{\partial }{\partial \tau })^{k} + i\gamma L|E|^{2} } ]E + \sqrt \theta E_{in} $$

where *T*_*R*_ is the roundtrip time of the cavity, $\alpha_{i}$ is the intrinsic loss per roundtrip of the cavity, and $\delta_{0}$ is the pump detuning, respectively. *β*_*k*_ = *d*^*k*^*β/dω*^*k*^ describes the *k*th-order dispersion coefficient of Taylor series expansion of the propagation constant at the pump frequency, *γ* is the Kerr nonlinearity coefficient, *L* is the circumference of the resonator, and *θ* is the coupling coefficient. *t* is the slow time describing the evolution of the intracavity field *E* (normalized such that the cavity power *P*_*cavity*_ =*|E|*^*2*^) while *τ* is the fast time describing the wave traveling at the group velocity in the resonator. *E*_*in*_ is the input pump field. The simulation parameters are set with intrinsic Q_in_ = 3 million ($\alpha_{i}$ = 0.0017), *β*_*2*_ = − 50 ps^2^/km, *β*_*k*_ = 0 for *k* ≥ 3 (higher order dispersion is ignored), γ = 1 W^−1^ m^−1^ (assuming effective area of waveguide *A*_*eff*_ = 1 μm^2^), and *L* = 2π (100 μm) with group index n_g_ = 2, unless mentioned otherwise. The free-spectral range (FSR) for the resonator is around 239 GHz. These parameters are based on traditional silicon nitride microresonators^5,14,16^ which is currently the most popular on-chip comb platform. Unlike the previous studies emphasizing the comb dynamics to the pump detuning^10,13,14^, our works here study the comb generation dynamics by varying the pump power. For integrated sources, although the wavelength tunability and linewidth reduction have been well-established by the control of external cavities and micro-heaters^22,23^, developments of fixed wavelength on-chip lasers with ultra-narrow linewidth^24^ are still advantageous for high-purity comb operation. This idea has been demonstrated by mode-locking solitons in microresonators with the aid of thermal tuning^25^. Nonetheless, the discussion on comb generation with a fixed pump detuning is still limited. A few literatures^13,14^ show the comb generation by varying the input power, but only a specific coupling case is studied. With the LLE, we would be able to model the comb threshold in relation to the pump power and cavity coupling while a locked, low-noise fixed-frequency laser is used as the pump.

## Results

### Comb generation dynamics for different couplings

**Figure 1:**
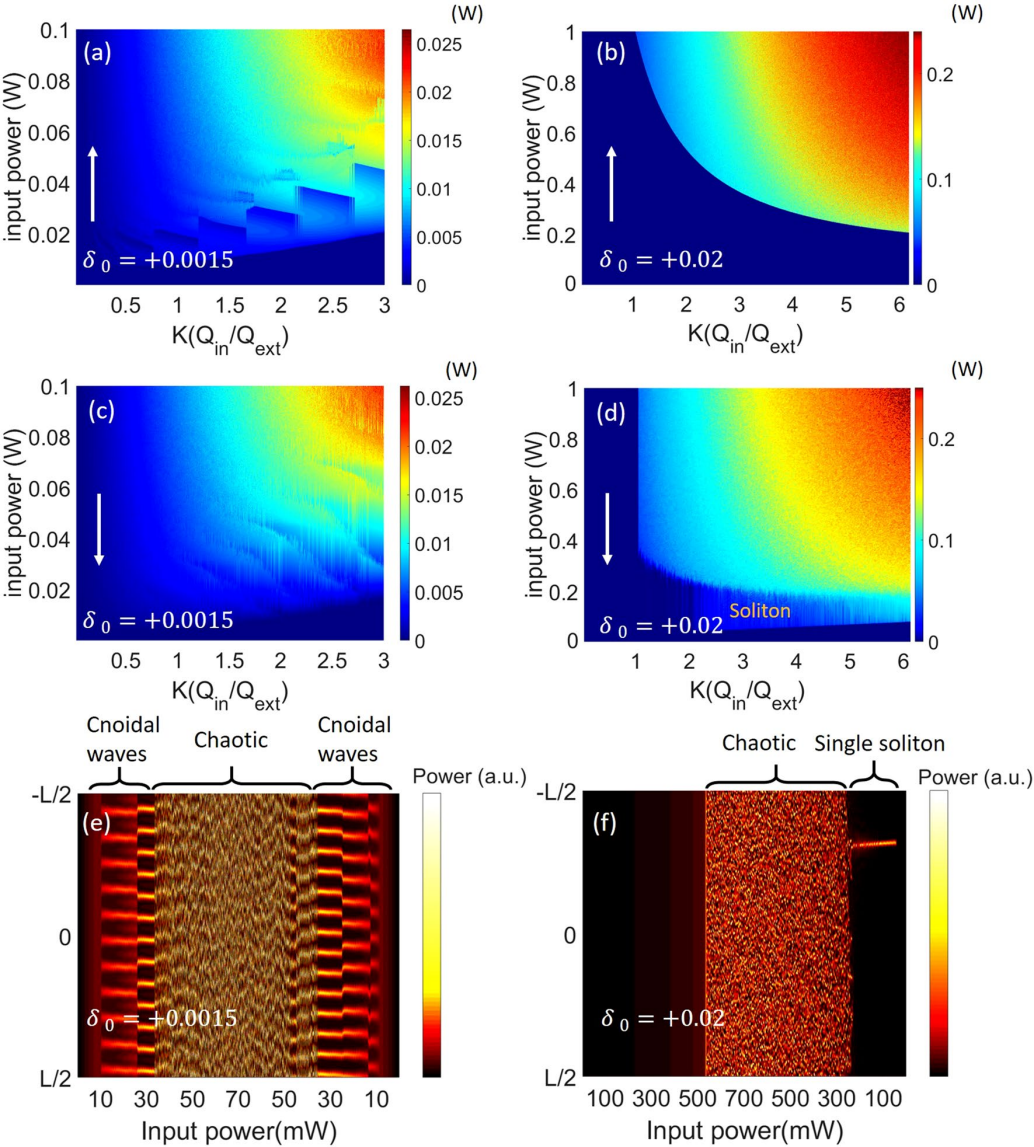
Comb output power in the parameter space of input power and coupling at detuning *δ*_*0*_ =  + 0.0015 and *δ*_*0*_ =  + 0.02 for (**a**, **b**) up-ramping the pump and for (**c**, **d**) down-ramping the pump. The color bar shows the total comb power at the output waveguide, excluding the pump line. (**e**, **f**) the corresponding evolution of cavity intensity as varying the input power with a fixed coupling factor *K* = 2. Stable cnoidal waves and a single cavity soliton are identified at detuning *δ*_*0*_ =  + 0.0015 and *δ*_*0*_ =  + 0.02, respectively.

In Fig. 1a,b, we show the simulation of the output comb power in the parameter space of coupling and input (bus-) power by up-ramping the input power, and in Fig. 1c,d, we show the similar mappings of the comb power by down-ramping the input power. The white arrows show the direction of power ramping. The exemplary pump detuning is set at *δ*_*0*_ =  + 0.0015 (frequency detuning = 57 MHz) in Fig. 1a,c and + 0.02 (frequency detuning = 760 MHz) in Fig. 1b,d, corresponding to a small/large detuning in comparison to the cavity linewidth. The coupling factor *K* is defined as the intrinsic Q (Q_in_)/external Q (Q_ext_). First, to characterize the cold-cavity threshold, we vary the input power at the bus waveguide slowly from zero and keep the pump detuning the same. This can be achieved by changing the current of the integrated semiconductor optical amplifier (SOA) and stabilizing the frequency with an integrated sensor^23^. The boundary of MI threshold is mapped by solving the LLE within the region of the coupling and input power. We can observe that the minimal point of the threshold is found at under-coupling (*K* = 0.5) with *δ*_*0*_ =  + 0.0015 in Fig. 1a but shifted to strong over-coupling (*K* = 11) with a large detuning *δ*_*0*_ =  + 0.02 in Fig. 1b. As stated in the previous literatures^8,26^, the threshold equation can be solved by equating the parametric gain and cavity loss per roundtrip:

2.1 $$ 2\gamma P_{cavity} \cdot L = 2\frac{{\omega n_{2} }}{{cA_{eff} }}P_{cavity} \cdot L = \frac{{n_{g} }}{c}(\frac{1}{{Q_{in} }} + \frac{1}{{Q_{ext} }}) \cdot L $$

2.2 $$ P_{th} (resonance) = \frac{{\pi n_{g}^{2} LA_{eff} }}{{4\lambda n_{2} Q_{in}^{2} }}\frac{{(1 + K)^{3} }}{K} $$

where *ω* is the pump (angular) frequency, *n*_*2*_ is the nonlinear refractive index, *c* is the speed of light in vacuum, *n*_*g*_ is the waveguide group index, and *λ* is the pump wavelength in vacuum. This equation assumes that the pump is at resonance peak and the threshold is optimized at under-coupling (*K* = 0.5). Once the pump frequency shifts away from the resonance, the cavity power reduces and the minimal threshold moves into the over-coupling regime^8^. Experimentally, the frequency shift could be introduced from either the thermally-induced resonance shift or pump detuning. Blue-to-red tuning method is used to achieve soft-thermal-locking and to maximize the circulating power for comb generation^2^. However, with high power in the cavity, the Kerr nonlinearity contributes to additional resonance shift toward the lower frequency regime. Before moving forward, we shed insights into the comb dynamics. Figure 1e,f show the cavity intensity first by up-ramping the input power with a fixed coupling factor *K* = 2, corresponding to Fig. 1a,b, and then by down-ramping the input power, corresponding to Fig. 1c,d. In Fig. 1e, comb power first grows into a stable (coherent) region with the rise of pump power. Periodic solutions of cnoidal waves are observed, similar to the earlier findings in the parameter space of pump power and detuning^13^. The stable region exhibits cnoidal waves with periodicities N in the range of 6 ~ 14 which corresponds to a Kerr comb with frequency spacing N times the microresonator FSR. While the pump gets higher, the periodicity of the cnoidal waves evolves, e.g. from N = 11 into N = 13 in Fig. 1e, between a stability boundary, resulting the ragged, step-like feature in Fig. 1a. As further up-ramping the power, the cnoidal waves eventually collapse into the chaotic region. Then, by tuning the input power backward, stable cnoidal waves with similar evolution of periodicities are observed again.

However, comparing Fig. 1f to e, the comb directly reaches the chaotic region without forming cnoidal waves as up-ramping the input power. The required input power for cavity MI increases from tens of mW to hundreds of mW. This behavior is explained by the unstable solution of MI branch^27,28^. For a small detuning, steady-state solutions of MI can be achieved by directly ramping up the input power; while for a large detuning, this process requires higher input power for switching the operation to the upper branch of the bistable hysteresis cycle^28^. As down-ramping the power, we can observe the generation of single cavity soliton. This initiation process, similar to that identified in Ref.^13^, is because of the satisfaction of cavity soliton by selecting detuning above the up-switching point^27^. Last, comparing Fig. 1d to b, hysteresis behavior is prominently identified for up- and down-ramps of the power. This is explained by operating at the upper branch of the bistablility curve. A boundary is now observed, corresponding to the transition from the chaotic waves to solitons. Due to the high peak power of the chaotic waves (hard excitation), it is not surprise that soliton solutions can exist in the regime where no MI is observed as increasing the pump power from the cold cavity. In addition, to verify the solution of single soliton in this system, we calculate the soliton number based on the nonlinear length *L*_*NL*_ = 1/*γP* and the dispersion length *L*_*D*_ = *τ*_*p*_^2^/|*β*_*2*_|, where *P* = 63 W is the soliton peak power and *τ*_*p*_ = 41 fs is the pulse duration. The evaluated soliton number $\sqrt{{L}_{D}/{L}_{NL}}$ = 1.5, which supports the operation regime of a single soliton.

### Parametric oscillation threshold from the LLE

**Figure 2:**
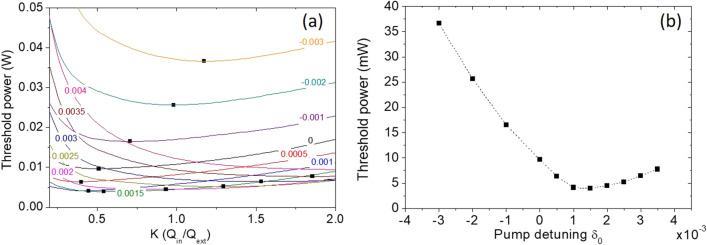
(**a**) Threshold power versus coupling for the pump detuning *δ*_*0*_ = − 0.003 to + 0.004. Each trace is obtained by solving the LLE individually. (**b**) Minimal threshold power versus the pump detuning, corresponding to the black dots in (**a**).

Now, we emphasize our findings in the threshold curves. To define the threshold in the LLE, the simulation is initiated by a random-noise intracavity field with a standard deviation 3 × 10^–9^ [W^1/2^], while the normalization *P*_*cavity*_ =|*E*|^2^ is equivalent to an average power of − 133 dBm. By increasing the input power, the threshold is then determined at which the parametric signal is 15 dB above the noise background from the simulated frequency spectra, in order to avoid catching unwanted background noise. Figure 2a shows threshold curves for different detunings from *δ*_*0*_ = − 0.003 to + 0.004, exhibiting similar tilt, U-shaped curves as identified in Fig. 1a,b. By varying the detuning, the minimal threshold of each curve (black dots) follows an asymmetrical trajectory which passes through the under-coupling regime with a relatively small detuning; then this trajectory extends to the over-coupling regime with a larger detuning. We also show the corresponding curve of the minimal threshold versus the pump detuning in Fig. 2b. Clearly, the minimal point occurs with a small but non-zero detuning around *δ*_*0*_ =  + 0.0015.

### Modified threshold equations from the traveling wave theory

To elaborate the identification from the LLE, we introduce the modified threshold equations from the traveling wave theory^29^. By including the phase shift, the threshold at the bus waveguide can be now expressed as follows:

3.1 $$ P_{cavity} = \frac{{\frac{\omega c}{{n_{g} L}} * (\frac{1}{{Q_{ext} }})}}{{(\omega - \omega_{0} )^{2} + \frac{{\omega^{2} }}{4}*(\frac{1}{{Q_{ext} }} + \frac{1}{{Q_{in} }})^{2} }}P_{bus} $$

**Figure 3:**
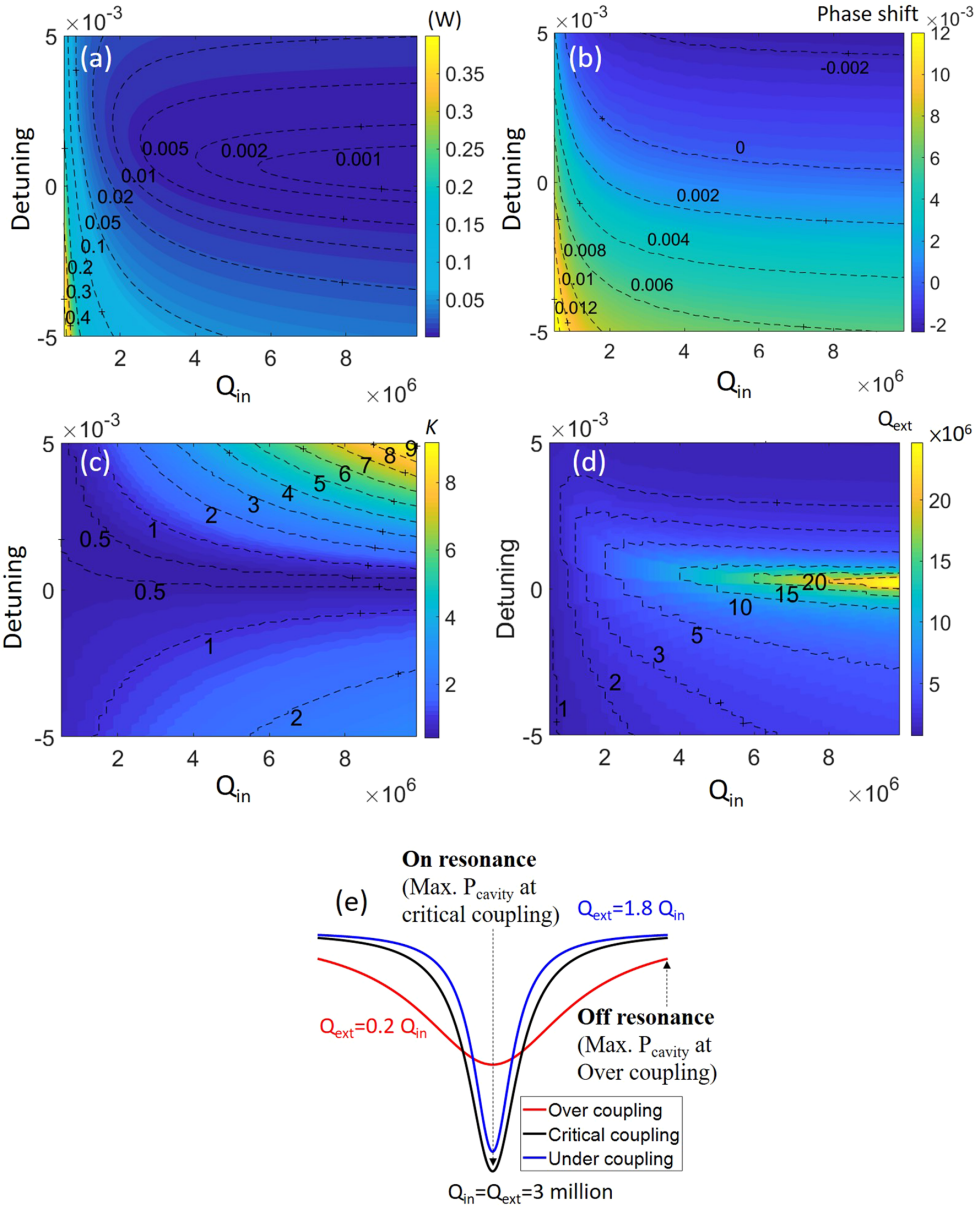
Results showing the prediction of (**a**) the threshold power, (**b**) the phase shift, (**c**) the coupling factor *K*, and (**d**) the external quality factor Q_ext_ from the traveling wave theory. (**e**) The schematic illustration of cavity resonances at different couplings.

3.2 $$ P_{th} = P_{th} (resonance) + \frac{{n_{g}^{2} LA_{eff} (\omega - \omega_{0} )^{2} }}{{2n_{2} \omega c}}(1 + \frac{1}{K}) $$

where $\omega - \omega_{0} = ( - \delta_{0} + \gamma L|E|^{2} )/T_{R}$ is the frequency shift as described in the LLE. Equation (3.2) is obtained by rearranging Eqs. (2.1) and (3.1). The second part on the right side of Eq. (3.2) suggests the additional power required for comb generation at the bus waveguide due to the frequency shift (off peak resonance). In Fig. 3a, we numerically solve the threshold equation in the parameter space of Q_in_ (from 10^5^ to 10^7^) and pump detuning (-0.005 to + 0.005). Comparing the results to the previous LLE solutions, we now extend the coupling dependency of the threshold power to different Q_in_. The phase shift, coupling factor *K*, and the Q_ext_ are also plotted in Fig. 3b–d, corresponding to those at the minimal threshold for a given Q_in_ and detuning. The phase shift shown here includes both the pump detuning (*δ*_*0*_) and Kerr-induced self-phase modulation (SPM).

First, we look at the relation between the threshold power and the cavity loss. Intuitively, for a given detuning, the threshold exhibits quadratic growth as the Q_in_ decreases. It reaches the minimum when the Q_in_ is high and the phase shift is weak ($- \delta_{0} + \gamma L|E|^{2} \approx {0}$). For the negative pump detuning, the phase shift adds up and results in large increase of the threshold power; on the other hand, for the positive detuning, it cancels out the phase induced by SPM. We can see that the curve of zero phase locates in the regime of the positive detuning in Fig. 3b. As Q_in_ decreases, more positive detuning is required—this is realized by the larger cavity power for comb initiation in a low-Q_in_ device.

Next, we consider the coupling condition, shown in Fig. 3c. Following the curve of zero phase shift in Fig. 3b, the minimal threshold power is found to be close to *K* = 0.5 as expected by Eqs. (2.1) and (2.2). However, with large phase shifts, the coupling is altered from *K* = 0.5 (under-coupling) to larger values, even up to 9 (strong over-coupling); the corresponding external quality factor (Q_ext_) is also mapped in Fig. 3d. To illustrate this coupling dependency, we show the scheme of cavity resonances at different couplings in Fig. 3e. In order to achieve the threshold condition, the optimized coupling with large phase shifts could be at over-coupling for better power enhancement than that at critical-coupling, especially for a high-Q_in_ resonator with a narrow intrinsic cavity linewidth. Meanwhile, this large positive detuning contributes to a negative phase shift in which the detuning surpasses the Kerr-induced SPM—the regime (effectively red detuned) where the cavity soliton is typically discussed^10^. Since the compensation between the positive detuning and SPM, the magnitude of the resulted phase shift is not as strong as that attributed from the negative detuning. Thus, the minimal threshold shows a gentler ramp-up rate. Take Q_in_ = 10 million cavity as an example, 15 times increase of the threshold power is observed at the detuning + 0.005 comparing to that at zero detuning; while 54 times increase is observed at the detuning − 0.005. We can also see the asymmetrical feature in Fig. 3c with respect to the curve of zero phase shift in Fig. 3b. For the phase shift with the same magnitude but opposite polarities (e.g. phase shift =  ± 0.002), the cavity power is not the same in each case due to the relative weighting of the Kerr-induced resonance shift and the pump detuning to the phase shift. This observation is unique in comparison with the traditional parametric oscillation threshold theory, considering the Kerr-induced frequency shift^8^.

We should note here that strong over-coupling will also degrade the total quality factor in cavities. Therefore, even with optimized coupling, the threshold power is raised up to an order of magnitude for a large detuning. In practical applications, high coupling efficiency is typically realized by optimizing the gap between bus waveguide and microresonators. Strong over-coupling with coupling factor *K* > 9 is possibly achieved^30,31^. However, for most of the cavities studied, the coupling factor *K* is compromised (*K* ≪ 9). It suggests an increase of threshold to reach the chaotic regime and later to initiate the cavity soliton, as previously identified in Fig. 1b.

### Threshold comparison between the LLE and the traveling wave theory

**Figure 4:**
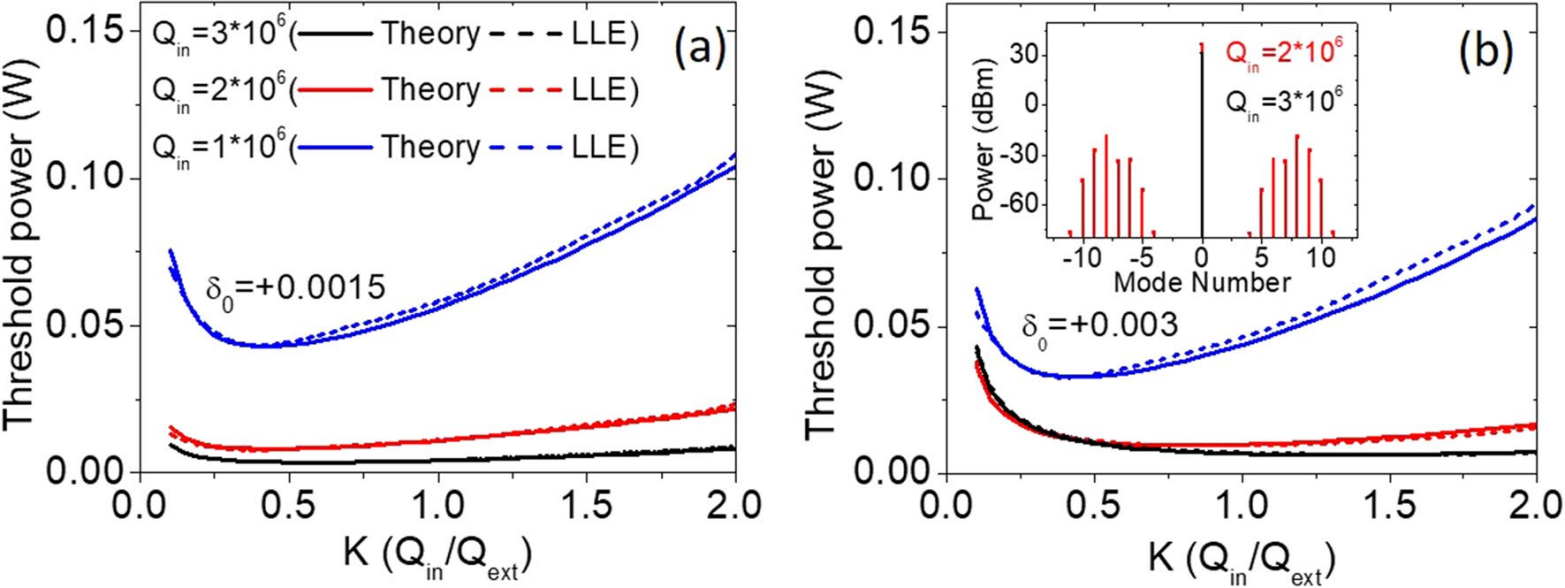
Simulated threshold for resonators with Q_in_ = 1, 2, and 3 million. The detuning is set at (**a**) *δ*_*0*_ =  + 0.0015 and (**b**) *δ*_*0*_ =  + 0.003. Solid lines stand for the simulated results from the traveling wave theory while the dashed lines stand for that from the LLE.

To further verify this theory, the LLE is again utilized to model the comb generation threshold. Figure 4 show the simulated threshold power with Q_in_ = 1, 2, and 3 million at *δ*_*0*_ =  + 0.0015 and + 0.003, both from the traveling wave theory (solid lines) and from the LLE (dashed lines). We can see that these data are in close agreement with each other, proving the validity of this model. The slight offset especially for a relatively low-Q device can be explained by the required power above the noise background in the LLE as mentioned earlier. Again, in comparison with Fig. 3d at a fixed detuning, the minimal threshold moves from under-coupling to over-coupling with increasing Q_in_. This effect is more notably for a larger detuning (e.g. *δ*_*0*_ =  + 0.003) as shown in Fig. 4b. We can clearly see that the minimal threshold moves from under-coupling (*K* ≈ 0.5 for Q_in_ = 1 million) to over-coupling (*K* ≈ 2 for Q_in_ = 3 million). With an even larger detuning, although not shown here, the optimized coupling of the minimal switching power at detuning *δ*_*0*_ =  + 0.02 in Fig. 1b is also verified by this model, showing the coupling factor *K* ≈ 11. Another interesting point is that, with a sufficient pump detuning, the required threshold power is similar for different Q_in_ at under-coupling; especially at strong under-coupling (*K* < 0.4), the threshold power for Q_in_ = 2 million is even lower than that for Q_in_ = 3 million. An exemplary comb spectrum in the cavity is shown in the inset of Fig. 4b. With coupling factor *K* = 0.2 and input power 20 mW, the comb is generated for Q_in_ = 2 million but not for Q_in_ = 3 million. This is because that, in order to satisfy the threshold condition, larger cavity power is needed for a low-Q device. The strong cavity power shifts the resonance by the Kerr nonlinearity and compensates the pump detuning. It therefore results in less phase shift and reduces the required power at the bus waveguide. This identification suggests that, for a preferable detuning, coupling design can be as critical as minimizing the cavity loss.

### Comb efficiency

**Figure 5:**
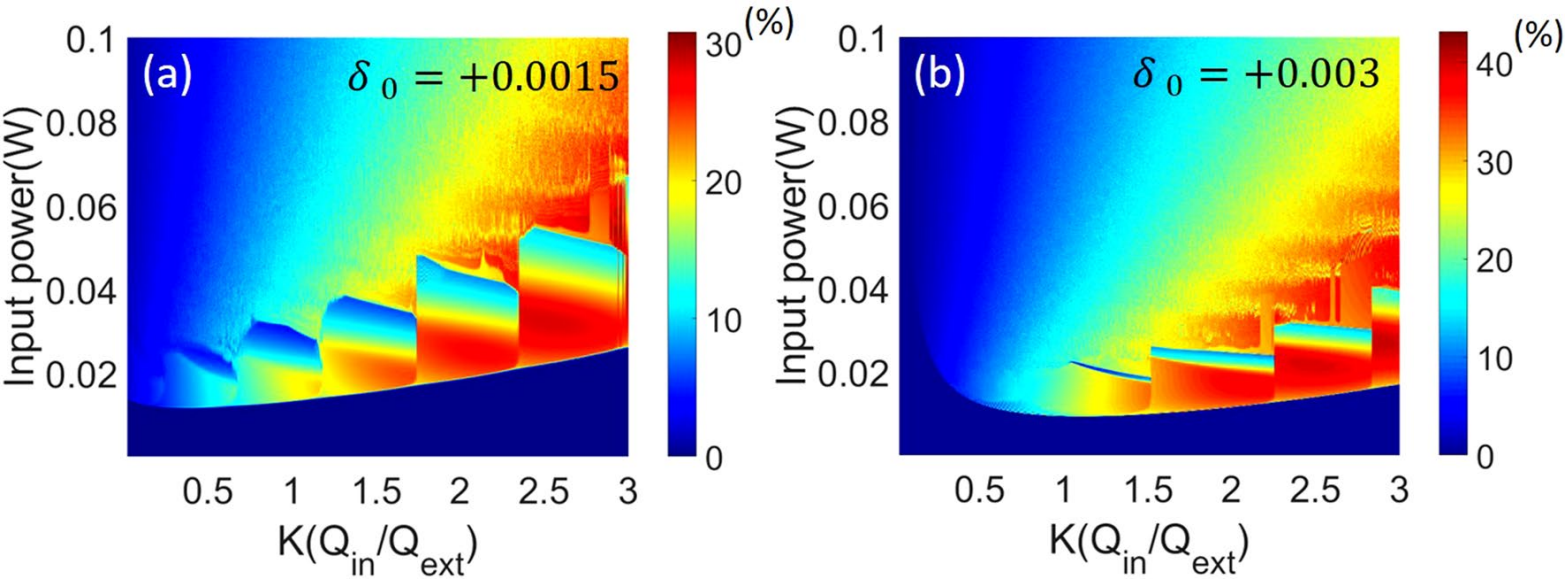
Conversion efficiency color-maps of the coupling and the input power at detuning (**a**) *δ*_*0*_ =  + 0.0015 and (**b**) *δ*_*0*_ =  + 0.003.

Finally, we look at the conversion efficiency of the input pump to all combs at the output waveguide. Figure 5 show efficiency color-maps at detuning *δ*_*0*_ =  + 0.0015 and + 0.003 by up-ramping the power from the cold cavity. Combs with efficiency up to 40% can be generated in the form of stable cnoidal waves from an over-coupled cavity, yielding higher efficiency than that from a critical-coupled cavity. This is in consistent with that observed from bright/dark soliton combs^16,18^; however, unlike the soliton case, the high efficient combs are now operated in the low-power, cnoidal wave regime. There are two reasons for the preference of over-coupling. First, as previously mentioned, the pump saturates in the cavity in the presence of comb formation as proposed in^17^. It moves the optimal coupling to an initially over-coupling regime. Second, stronger coupling also enhances the transfer of the comb power from the microresonator to the output waveguide.

**Figure 6:**
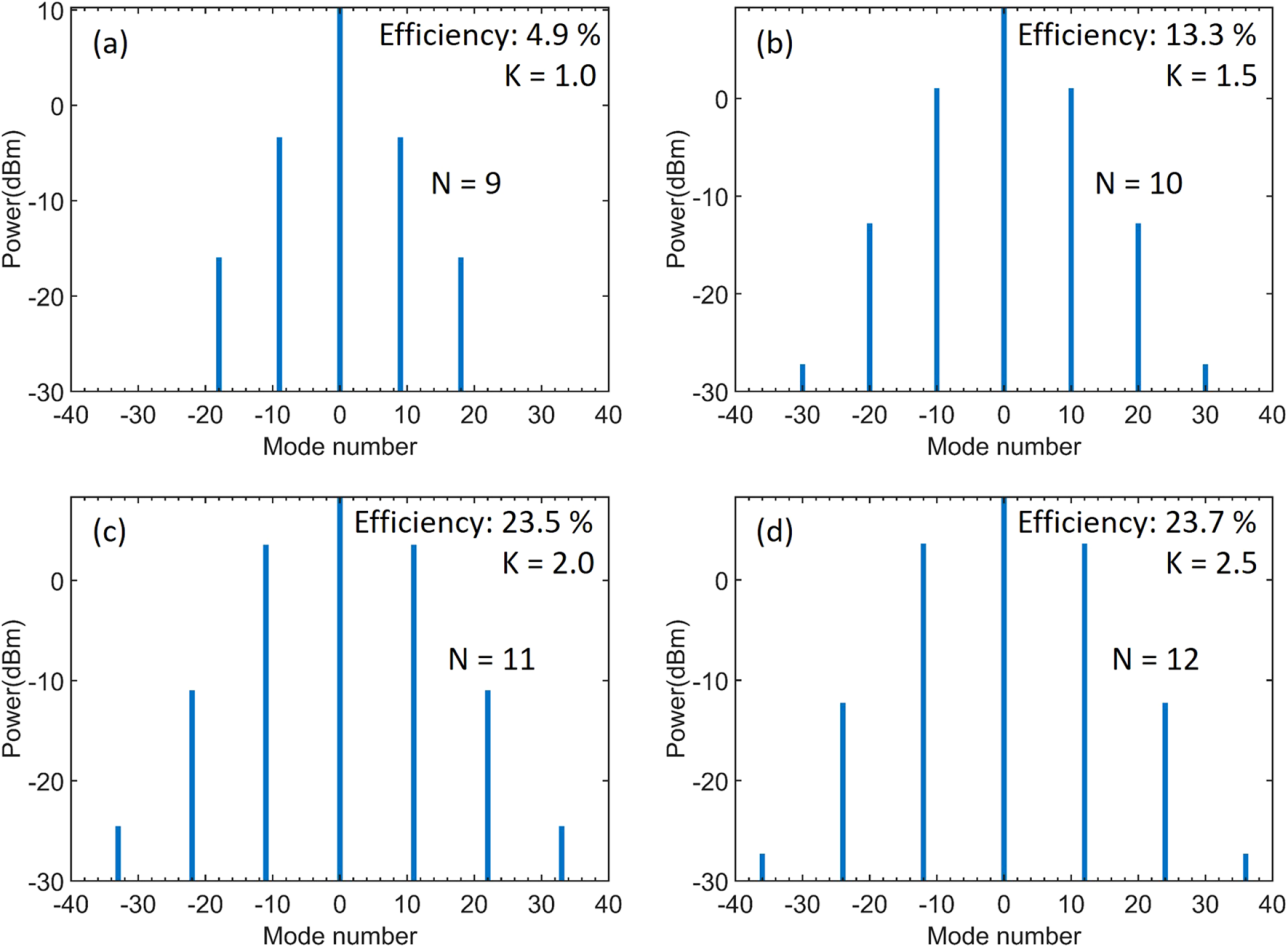
Comb spectra with input power = 20 mW and detuning =  + 0.0015. The coupling factor is set at (**a**) *K* = 1, (**b**) *K* = 1.5, (**c**) *K* = 2, and (**d**) *K* = 2.5, respectively.

**Figure 7:**
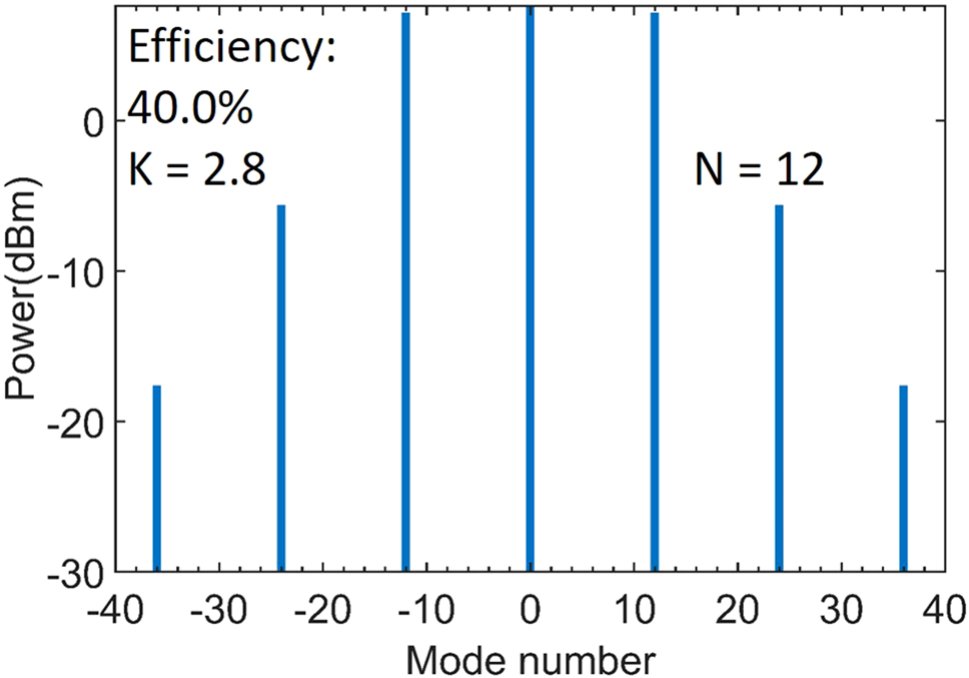
Comb spectra with 40% efficiency for detuning =  + 0.003, input power = 28 mW, and coupling factor *K* = 2.8.

Meanwhile, step-like boundaries can be observed above the threshold in the color-maps. These boundaries correlate to cnoidal waves with different periodicities N. Since the cnoidal wave solutions with different periodicities can be stable at the same point near the boundary, this evolutionary solution may yield ambiguous results in the adjacent step regions^13^. To further study the comb dynamics, we show the exemplary output spectra in Fig. 6 for *K* = 1 to 2.5, detuning *δ*_*0*_ =  + 0.0015, and input power = 20 mW. Comparing these results, we can see that the periodicity N increases from 9 to 12, corresponding to increasing frequency spacing of the combs, as the coupling factor *K* increases. The conversion efficiency is found to be enhanced from 4.9 to 23.7%. It has been shown that for a soliton-comb with a fixed FSR, the conversion efficiency is inversely proportional to the number of comb lines^16^, while for cnoidal waves with a N-periodic train, the efficiency is nearly proportional to the periodicities N^13^, which is qualitatively consistent with our findings here. Based on these results, for any given detuning, the optimized efficiency can be found in the parameter space of power and coupling. Figure 7 shows the comb spectrum with 40% efficiency for detuning *δ*_*0*_ = 0.003, input power = 28 mW, and coupling factor *K* = 2.8. Furthermore, despite the periodicity, the conversion efficiency also correlates to the coupling in the same step region, as previously shown in Fig. 5. While we modify the factor *K* from 2.8 to 3.4, the efficiency drops to 34.6% with the same periodicity N = 12. Using this map, we can optimize the coupling power and initiate efficient combs for low-power operation. To compare the solution of a bright pulse, we also compute the efficiency for the single soliton case in Fig. 1d, only 5.6% efficiency is seen even at over-coupling, a finding confirmed by simulation in^15,16^.

Last, we analyze the dependence of periodicity on the coupling. As discussed above, the intracavity power is dependent on the coupling and detuning, determining the position of the maximum MI gain (at frequency $f_{\max } = \frac{{\sqrt {2\gamma P_{cavity} /|\beta_{2} |} }}{2\pi }$ from the pump frequency)^32^. Therefore, the periodicity of primary comb generation can be evaluated by N = $f_{\max } /FSR$. We take Fig. 6d as an example—with detuning *δ*_*0*_ =  + 0.0015 and *K* = 2.5 (Q_ext_ = 1.2 million), the intracavity power at pump is 7.6 W, resulting in the periodicity of the primary comb N ≈ 12. This agrees well with the simulated spectrum. Interestingly, compared to that in Fig. 6a with *K* = 1, the periodicity decreases to N = 9 with even less cavity loss (Q_ext_ = 3 million), which seems to contradict the relation above ($N \propto \sqrt {P_{cavity} }$). We should emphasize here again that, with non-zero phase shift, critical-coupling does not guarantee the largest build-up power in the cavity. For *δ*_*0*_ =  + 0.0015, the intracavity power at pump with *K* = 1 is actually less than that with K = 2.5 and thus results in the decrease of the periodicity. As for the fixed coupling and detuning, the periodicity increases when increasing the input power as shown in Fig. 1c, which is in consistent with the theory. Here, our work provides additional information of coupling on the periodicities of cnoidal waves.

## Discussion

We should point out, although strong over-coupling is applied in the LLE model, the external Q (Q_ext_) is still high (> 10^5^) in our analysis when the order of the discussed phase shift is < 10^–2^. Thus, it satisfies the mean-field assumptions that the field change over a single roundtrip is weak, asserting the validity of the LLE^9,12^. Furthermore, unlike the LLE, the threshold power evaluated based on the traveling wave theory is assumed to be independent to the fast-time temporal profile of the intracavity field. This assumption is valid while the pump power is below and close to the threshold power, resulting constant intensity in the cavity. This process can also be explained by replacing a CW for the cavity field in the LLE. The relation between detuning, threshold power, loss, and coupling is naturally analogous to the traveling wave theory. Therefore, for CW approximation, although we ignore the higher-order dispersion, the dispersion parameter has no contribution to the simulated threshold curves. We have verified this by replacing the dispersion parameter *β*_*2*_ from − 10 to − 100 ps^2^/km which are achievable values by waveguide engineering and no difference in threshold curves is identified.

**Figure 8:**
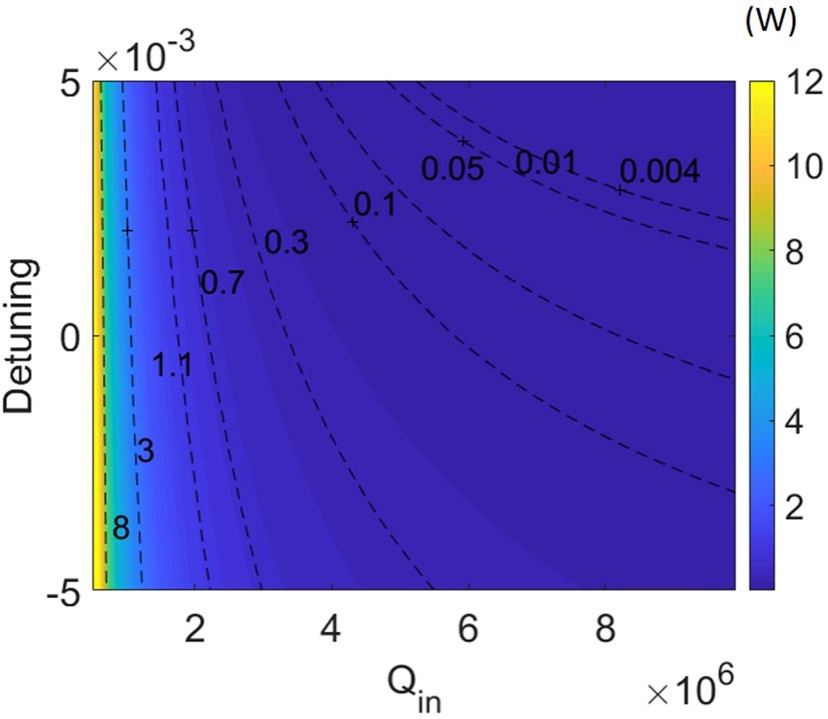
Color-map of the threshold power with the thermal induced shift $\gamma_{T} = 10\gamma$.

In addition, although we do not take into account the thermally induced resonance shift which could be more significant than SPM, this red-shift effect could be approximated to be proportional to the intracavity power^33^. Therefore, the frequency shift is expressed as:

4 $$ \omega - \omega_{0} = ( - \delta_{0} + \gamma L|E|^{2} + \gamma_{T} L|E|^{2} )/T_{R} $$

where $\gamma_{T}$ is the coefficient for the thermally induced shift which could be typically 5 to 10 times higher than $\gamma$^33,34^. We can use this approximation, at least qualitatively, to explain the thermally resulted resonance shift. Since both the Kerr nonlinearity and thermo-optic effect red-shift the resonance, this simplification shows a stronger power-dependent shift for the analysis above. Figure 8 shows the exemplary map of the threshold power by including the thermal induced shift $\gamma_{T} = 10\gamma$. Clearly, as compared to that in Fig. 3a, more positive detuning is required to compensate the thermal induced shift for the minimal threshold. Meanwhile, the thermal-optic effect may also introduce thermal disturbance to the hybrid-integrated laser source when either to tune the wavelength or change the output power; accurate frequency control can be developed by integrated on-chip sensors^23^.

Last, in addition to the silicon nitride platform discussed here, this finding can be applied to different materials or resonator-based cavities for chip-based comb generation. Unlike the coupling in integrated devices, suspended geometries, such as tapered fibers and prisms, have been widely used in wedges, micro-toroid, and crystalline resonators. Although these free-standing couplings offer a more flexible way to optimize the coupling, it requires complicated optical alignment and also limits the device density^35^. For the integrated platform, a lithographically patterned bus waveguide overcomes these difficulties but the coupling strength is restricted by the fabrication capability. Therefore, for planar resonators with a compromised Q, the design of efficient coupling, such as a pulley-coupled bus waveguide used in silicon nitride^31^ and gallium nitride^36^ platforms, is desirable to achieve over-coupling for high efficient comb generation.

## Summary

To conclude, we have determined the threshold boundary for comb generation within the parameter space of pump power and coupling by solving both the LLE and the traveling wave theory. The minimal position of the threshold power exhibits a strong dependence on the cavity coupling. We numerically show that, with a large phase shift, the coupling is optimized to be strongly over-coupled; while with zero phase shift, the optimized point moves to the under-coupled regime. In comparison with the previous methods, our work reveals the relation between pump detuning, coupling, and comb dynamics. In addition, the comb evolution is studied from a fixed wavelength approach. Cnoidal waves and solitons are both demonstrated by varying the input power at different couplings. It also evidences that the coupling design can be more critical than the intrinsic quality factor Q_in_ under specific conditions. Besides, the relation between comb efficiency and coupling is discussed, especially around the threshold. Unlike the previous discussion on bright / dark cavity solitons, high-efficient combs in the form of cnoidal waves are observed with less required power at the input. Our numerical results provide a promising pathway to design chip-based cavities with optimized coupling, especially for systems requiring low-power operation and high-efficient (coherent) microcombs.
